# Preclinical Lymph Node Model for Intraoperative Molecular Imaging of Cancer

**DOI:** 10.21203/rs.3.rs-2953015/v1

**Published:** 2023-06-12

**Authors:** Patrick Bou-Samra, Austin Chang, Sachinthani Arambepola, Emily Guo, Feredun Azari, Gregory Kennedy, Alix Segil, Sunil Singhal

**Affiliations:** University of Pennsylvania Perelman School of Medicine; University of Pennsylvania Perelman School of Medicine; University of Pennsylvania Perelman School of Medicine; University of Pennsylvania Perelman School of Medicine; University of Pennsylvania Perelman School of Medicine; University of Pennsylvania Perelman School of Medicine; University of Pennsylvania Perelman School of Medicine; University of Pennsylvania Perelman School of Medicine

**Keywords:** lymph node model, intraoperative molecular imaging, cancer imaging

## Abstract

**Purpose:**

Lymph node(LN) dissection is part of most oncologic resections. Intraoperatively identifying a positive LN(+ LN), that harbors malignant cells, can be challenging. We hypothesized that intraoperative molecular imaging(IMI) using a cancer-targeted fluorescent prober can identify + LNs. This study aimed to develop a preclinical model of a + LN and test it using an activatable cathepsin-based enzymatic probe, VGT-309.

**Procedures:**

In the first model, we used peripheral blood mononuclear cells (PBMC), representing the lymphocytic composition of the LN, mixed with different concentrations of human lung adenocarcinoma cell line A549. Then, they were embedded in a Matrigel^®^ matrix. A black dye was added to mimic LN anthracosis. Model two was created using a murine spleen, the largest lymphoid organ, injected with various concentrations of A549. To test these models, we co-cultured A549 cells with VGT-309. Mean fluorescence intensity(MFI) was. An independent sample t-test was used to compare the average MFI of each A549:negative control ratio.

**Results:**

A significant difference in MFI from our PBMC control was noted when A549 cells were 25% of the LN (p = 0.046) in both 3D cell aggregate models-where the LNs native parenchyma is replaced and the one where the tumor grows over the native parenchyma. For the anthracitic equivalents of these models, the first significant MFI compared to the control was when A549 cells were 9% of the LN (p = 0.002) in the former model, and 16.7% of the LN (p = 0.033) in the latter. In our spleen model, we first noted significance in MFI when A549 cells were 16.67% of the cellular composition.(p = 0.02)

**Conclusions:**

A + LN model allows for a granular evaluation of different cellular burdens in + LN that can be assessed using IMI. This first exvivo + LN model can be used in preclinical testing of several existing dyes and in creating more sensitive cameras for IMI-guided LN detection.

## Introduction

Although initially described by Hippocrates in the 5th century BC, Thomas Bartholin was the first to describe the entire human lymphatic system in 1652. Dr. Halsted underscored the clinical significance of nodes in cancer surgery by performing a radical mastectomy and harvesting its corresponding nodes to fully excise the cancer.[1] Nowadays, LN excision has become the standard of care in most oncologic resections and provides important therapeutic and diagnostic data. LNs can help in cancer staging, predict prognosis, improve overall survival, and improve disease-free survival.[2–4]

There has been an ongoing debate among surgeons on the better approach to harvest LNs for many cancer types such as lung, breast, pancreatic, colon, and prostate cancers. For example, in lung cancer surgery, some surgeons advocate for a lobe-specific approach, whereby LNs that are contiguous to the resected organ are harvested. However, 6% of patients in one study had missed positive nodes as they were in non-contiguous regions, thereby depriving these patients of adjuvant systemic treatment. Others follow a targeted approach to LN dissection like the omission of particular stations in the surgical resection of non-small cell lung cancer.[5] Some even rely on the tumor maximum standard uptake value (SUVmax) whereby a patient with a higher SUVmax likely has a more aggressive tumor and will benefit from a systematic dissection as opposed to a lobe-specific approach.[6] However, the most recent consensus is performing a systematic LN dissection. As per the European Society of Thoracic Surgeons (ESTS), at least 3 mediastinal lymph node stations, that always include station 7 (subcarinal area), station 10 (hilar lymph nodes) and 11 (interlobular lymph nodes), should be systematically dissected.[7] All of these guidelines highlight one of the major challenges intraoperatively in surgical oncology which is identifying a LN which harbors cancer cells, termed a “positive LN”.

The ultimate goal is to localize and dissect all positive LNs and help avoid leaving a false negative LN behind. One study described the incidence of false negatives to be 13.2% in systematic lymph node dissection of 219 patients with non-small cell lung cancer.[8] A non-specific aggressive dissection of all lymph node stations might help reduce false negatives. However, that procedure is associated with the morbidity of disrupting lymphatic channels, lymphedema, and increasing the magnitude of the surgery and injuring adjacent structures such as nerves and ducts.

We hypothesized that using intraoperative molecular imaging (IMI), we would be able to identify true positive LNs intraoperatively and decrease the false negative LNs. This allows for a more targeted lymph node dissection. In IMI, a fluorescent contrast-agent is injected into patients prior to surgery. It targets cancer cells which are then imaged with a camera that can detect the wavelength emitted by the dye.[9] Anecdotally however, there are patients who have positive LNs and still do not fluoresce intraoperatively. We suspect that the extent of tumor burden of a LN is positively correlated to its fluorescence.

As such, we begin by describing the first preclinical positive LN model with various concentrations of cancer cells. This model can serve as a template to test several cell lines and eventually contrast agents in the future using IMI. Secondly, we aimed to quantify the required cancer cell burden in a LN for it to fluoresce on IMI. We used cathepsin activity as a surrogate for cancer cell burden. Cathepsins are enzymes that are differentially overexpressed in tumor cells and often secreted into the extracellular space to promote tumor cell migration.[10, 11] The contrast agent we used was VGT-309 and is an activated-based probe that is cathepsin-activated. It has been previously shown to be able to selectively identify cancer cells.[12] As such, we postulated that using VGT-309 in positive LN that have cancer cells and hence higher cathepsin activity will allow us to visualize these nodes using IMI principles.

## Methods

### Cell Line

The human bronchoalveolar carcinoma cell line A549 was chosen for our model development given its previous use in studies of cathepsin expression in human lung cancer.[13] This cell line was maintained *in vitro* using R10 media containing RPMI, 10% fetal bovine serum (FBS), 2 mmol/L glutamine, and 5 mg/mL penicillin/streptomycin. It was regularly tested and maintained negative for *Mycoplasma spp*.

### Isolation of Peripheral Blood Mononuclear Cells (PBMC)

Peripheral blood mononuclear cells (PBMCs) are mononuclear cells (lymphocytes, monocytes, granulocytes, and platelets) extracted from the buffy coat, which makes up 1% of whole blood. These cell types constitute the bulk of the parenchyma of lymph nodes, which prompted their use in building our exvivo lymph node model.[14]

Blood samples were collected from patients undergoing lung resection for a suspicious lesion at the University of Pennsylvania. Samples were washed with Phosphate-Buffered Saline (PBS). Lymphoprep^®^, a density gradient medium, was added to allow layering and isolation of mononuclear cells. We centrifuged the solution and isolated the buffy coat layer. PBMCs were extracted and counted after washing the buffy coat with media containing Dulbecco′s Modified Eagle′s Medium/Nutrient Mixture F-12 Ham, 10% Cytiva HyClone Fetal Bovine Serum, 2 mmol/L glutamine, and 5 mg/mL penicillin/streptomycin. The number of PBMCs used in each model varied due to varying blood quantity and PBMC isolation yield on different days.

### Creation of a 3D cell-Aggregate as an Ex-Vivo Positive Lymph Node Model

**Figure 1:**
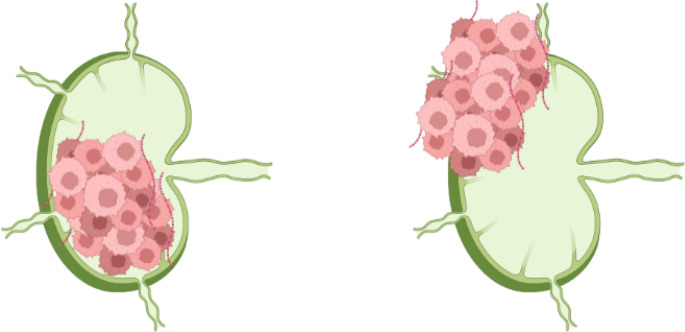
(left) LN model that assumes tumor replaces parenchyma (right) LN model with tumor growing over native parenchyma

A549 cells were added to PBMCs to form increasing ratios of A549:PBMC (1:10, 1:9, 1:8, 1:7, 1:6, 1:5, 1:4, 1:3, 1:2, 1:1, 2:1). A549 and PBMC pellets were resuspended in a 1:1 solution of PBS and lactose dehydrogenase elevating virus (LDEV)-free Matrigel Basement Membrane Matrix and transferred to a cylindrical mold (1 cm × 1 cm × 0.6 cm). Solutions were incubated at 37 °C for 30 minutes to facilitate aggregation. We used two different models with different assumptions. Our first model assumes that the total number of cells is the same and the intrinsic ratio of cancer to normal parenchyma changes as the tumor encroaches on native parenchyma. This is the case with renal cell carcinoma.[15] The other model assumes that the tumor grows in the lymph node and adds to the total number of cells present. (Fig. 1) FD&C Black No. 1 (CI 28440) (Black food coloring) was added to a portion of the 3D cell aggregates to account for the anthracosis of some lymph nodes that is seen in smokers. This is secondary to anthracitic particles that deposit in the lymph node of smokers.[16] The 3D cell aggregates were then imaged on the Pearl^®^ Trilogy (LI-COR Biosciences, Lincoln, NE).

### Using Murine Spleen as an Ex-Vivo Positive Lymph Node Model

#### Quantifying splenocytes in a murine spleen

C57BL/6 mice were sacrificed, and spleens were extracted for our experiment. Spleens were weighed and minced into small pieces (< 0.1 cm^2^) with a scalpel blade. Spleen pieces were resuspended in PBS and vortexed to further mince down. The excised spleen was transferred to a 30 μm pre-separation filter (Miltenyi Biotec, Bergisch Gladbach, Germany) for straining. The specimen was washed several times with PBS and ground up further to improve yield. Following a final centrifuge and PBS wash step, the specimen was resuspended in PBS and counted on the Countess II FL (Thermo Fisher, Waltham, MA).

#### Inoculating murine spleens with A549 cells

Murine spleens were split into thirds and weighed to quantify the number of splenocytes using the previously described method. A549 cells were injected into intact murine spleens in the same ratios described in our 3D cell aggregate models (A549:splenocytes – 1:10, 1:9, 1:8, 1:7, 1:6, 1:5, 1:4, 1:3, 1:2, 1:1, 2:1). Murine spleens were then imaged on the Pearl^®^ Trilogy (LI-COR Biosciences, Lincoln, NE).

#### VGT-309: quenchable activity-based probe

VGT-309 (chemical formula C_127_H_142_ClF_4_N_10_Na_3_O_23_S_5_; molecular weight, 2,517.29 Da) is a quenchable activity-based probe (qABP) that consists of a phenoxymethyl ketone electrophile that covalently and irreversibly binds active cysteine cathepsins, coupled to an ICG fluorophore (excitation peak = 789 nm, emission peak = 814 nm) and a IRDye QC-1 quencher (LI-COR Biosciences, Lincoln, NE). Cathepsin binds to the probe and cleaves the quencher, allowing fluorescent signals to be detected by NIR imaging systems. Vials containing 11 mg of lyophilized VGT-309 drug product were reconstituted with water for injection to a final concentration of 5 mg/mL and diluted with pH 7.4 PBS, culture media, or Baxter Compound Sodium Lactate (Hartmann’s) for the appropriate application[17] Tumor cells express an increased levels of cathepsin and when co-cultured with VGT-309 have been shown to activate the dye and fluoresce.[13]

##### Evaluation of In Vitro Binding and Fluorescence Signal Intensity of VGT-309 in A549 Cells by Fluorescence Microscopy

For confirmation of in vitro binding, A549 cells were cultured on poly-L-lysine-coated glass coverslips in 6-well plates with R10 media for 24 hours. Cells were incubated with 1 *μ*M VGT-309 for 2 hours at 37 °C. Coverslips were removed from culture following VGT-309 treatment, mounted on glass slides with ProLong Gold Antifade reagent with DAPI (Fisher Scientific, Waltham, MA), and imaged on a Leica DM6 B fluorescence microscope (Leica Microsystems, Wetzlar, Germany).

While keeping the dosage constant at 1 *μ*M VGT-309, we evaluated the correlation between fluorescence intensity and cathepsin activity (number of A549 cells). PBMCs (285,000 per sample) were mixed with an increasing number of A549 cells labeled with VGT-309: 300, 3000, 30000, 140000, 290000, and 540000 A549 cells. Cell mixtures were spun down into pellets and imaged on the Odyssey Imaging System (LI COR Biosciences, Lincoln, NE).

#### Testing of 3D Cell Aggregate and murine spleen models for detection by IMI

A549 cells were incubated with 1 *μ*M VGT-309 for 2 hours at 37 °C. Following VGT-309 treatment, cells were rinsed with PBS and added to PBMCs to form increasing ratios of A549:PBMC (1:10, 1:9, 1:8, 1:7, 1:6, 1:5, 1:4, 1:3, 1:2, 1:1, 2:1) in the 3D Cell Aggregate model.

For the murine spleen model, following VGT-309 treatment, A549 cells were rinsed with PBS and injected into intact murine spleens in the same ratios described in our 3D cell aggregate model. Then both models were imaged on the Pearl^®^ Trilogy (LI-COR Biosciences, Lincoln, NE). Throughout these experiments, A549 cells are a surrogate of cathepsin activity that is increased in cancer cells.[13]

### Post Hoc Image Analysis

Post hoc image analysis was conducted with ImageJ (http://rsb.info.nih.gov/ij). Fluorescence was quantified via mean fluorescence intensity by analyzing monochromatic NIR images and measuring regions of interest. Calculations were performed in triplicate.

### Statistical Analysis

In understanding the effect of increased cell count in the presence of constant dye concentration, we used the Pearson correlation coefficient. In comparing the different values among the varying concentrations, each concentration was compared to the negative control using an independent sample t-test. The average MFI of 3 repeated trials of each experiment was used in our calculations. The data was analyzed using IBM SPSS Statistics software version 28 (IBM Corp., Armonk, NY, USA).

## Results

### Ex-vivo lymph node Model 1: 3D cell aggregates of A549 and PBMCs

The lymph node is composed of several lymphoid lobules that are arranged side-by-side, each having a superficial cortex, paracortex, and a nodal medulla. In these lobules, there are several B and T lymphocytes, their cellular precursors, macrophages, and antigen presenting cells (APCs). The infrastructure of the lymph nodes is essentially the reticular meshwork, a family composed of fibroblast reticular cells. The Matrigel^®^ matrix was the reticular meshwork analogue we used. Lymphocytes are the bulk of the parenchymal cells of the lymphatic lobules.[14] The PBMCs constituted the cellular composition of the lymph node.

**Figure 2:**
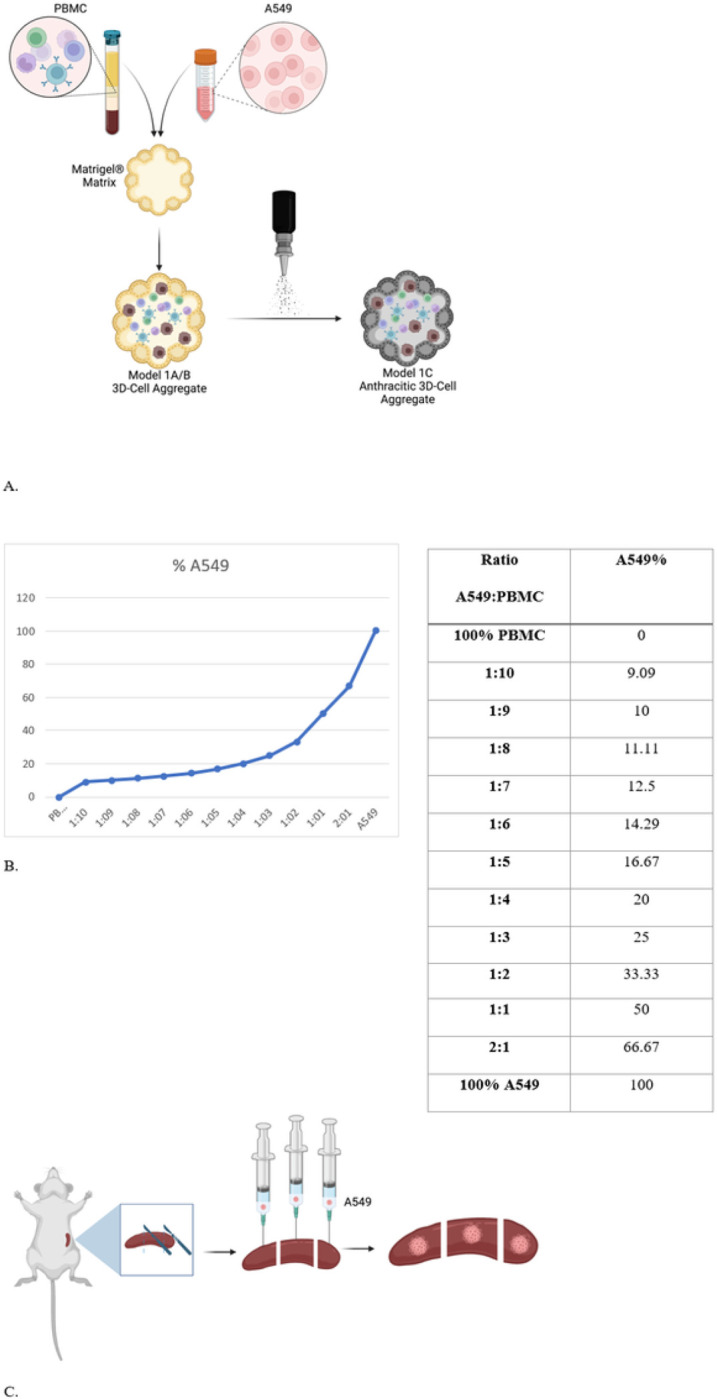
*Exvivo* positive LN models (A) Model 1: A/B 3D cell aggregates formed from a Matrigel^®^ matrix with PBMCs and A549 cells. Model 1A has increasing ratios of A549:PBMCs while keeping total cell count constant. Model 1B has increasing ratios of A549:PBMCs while keeping PBMC count constant for each ratio. To represent an anthracitic nodule of models 1A and 1B, black dye is added to the matrix in model 1C. (B) Varying ratios of PBMCs and A549 with the corresponding A549 percentage composition that is an analogue for cathepsin activity (C) Model 2: Murine model of sacrificed mice, spleen sectioned into 3 pieces, and injection of A549 into each piece.

- Model 1A: Increasing A549:PBMC ratios with a constant total number of cells to represent tumor replacing native lymph node parenchyma
  We first modeled LN in which tumor cells replace native parenchyma by keeping total cell count (A549 and PBMC) constant (312,500 cells) in our 3D cell aggregates. We gradually increased the percentage of A549 cells. (Fig. 2A/B)
- Model 1B: Increasing A549:PBMC ratio with a constant amount of PBMC to represent tumor growing adjacent to native lymph node parenchyma
  We then modeled a LN in which tumor cells proliferate on top of native parenchyma by keeping the number of PBMCs constant (160,000 cells) and increasing the proportion of A549 cells labeled with VGT-309. (Fig. 2A/B)
- Model 1C: Anthracitic LN model of 1A and 1B
  To mimic anthracitic LNs, we added FD&C Black No. 1 (CI 28440) to the models 1A and 1B. We then performed the same experiments whereby the A549:PBMC ratios were changed accordingly. (Fig. 2A)

### Ex-vivo Lymph Node Model 2: Inoculating Murine Spleens with A549 cells

After performing the quantification technique of splenocytes described earlier, we found that the splenocyte count was estimated to be 2.54 × 10^8^ splenocytes/gram.

To evaluate the use of murine spleens as an *ex vivo* model of positive lymph nodes, we calculated the number of splenocytes based on weight and injected spleen parenchyma with VGT-309-labeled A549 cells in increasing ratios. (Fig. 2C)

### Model Testing with IMI using VGT-309

#### VGT-309 fluorescence intensity is positively correlated with cathepsin activity in human bronchoalveolar carcinoma

**Figure 3:**
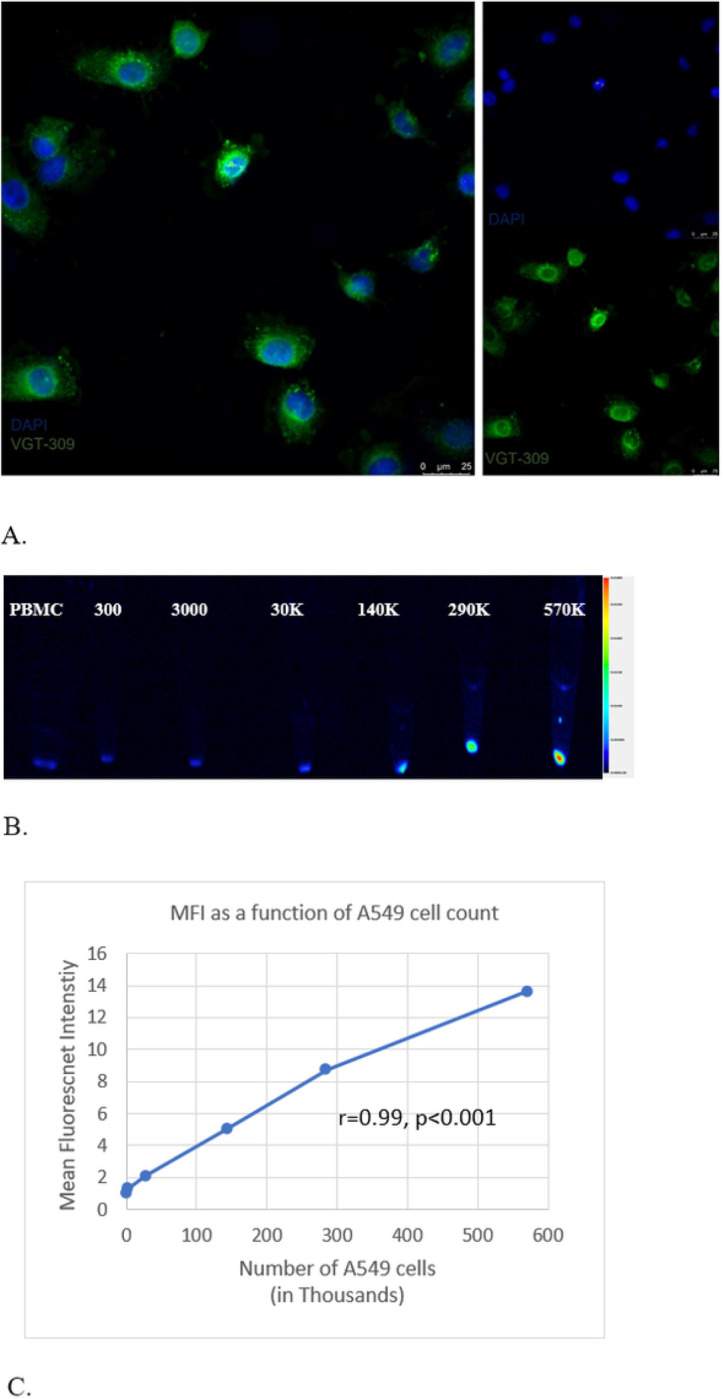
VGT-309 labels A549 cells in a cathepsin activity-dependent manner (A) Fluorescence microscopy of A549 (human pulmonary adenocarcinoma) cells at 40X magnification 2 hours after treatment with 1 μmol/L VGT-309. Cells were co-stained with DAPI. Overlay images are shown on left with DAPI and VGT-309 channels on right. Scale bars represent 25 μm. (B) Fluorescence signal intensity of A549 cells following 1 μM VGT-309 treatment. Negative control of PBMCs alone (285,000 cells) on left. Number of A549 cells per condition shown on top row.(C) MFI is positively correlated with cathepsin activity, indicated by the A549 cell count (R=0.99, p<0.001).

We first confirmed that VGT-309 is internalized and fluoresced in the presence of A549 cells (Fig. 3A). While keeping VGT-309 dosage at 1 μM and PBMC count at 285,000 cells, we observed a positive correlation between fluorescence intensity (MFI) and cathepsin activity (A549 cell count) r = 0.99 (p < 0.001), indicating that signal intensity is positively correlated with number of cancer cells and hence cathepsin activity. (Fig. 3B,C)

##### Model 1 Testing with VGT-309: 3D cell aggregates of VGT-309-labeled A549 and PBMCs

###### a. Model 1A

**Figure 4:**
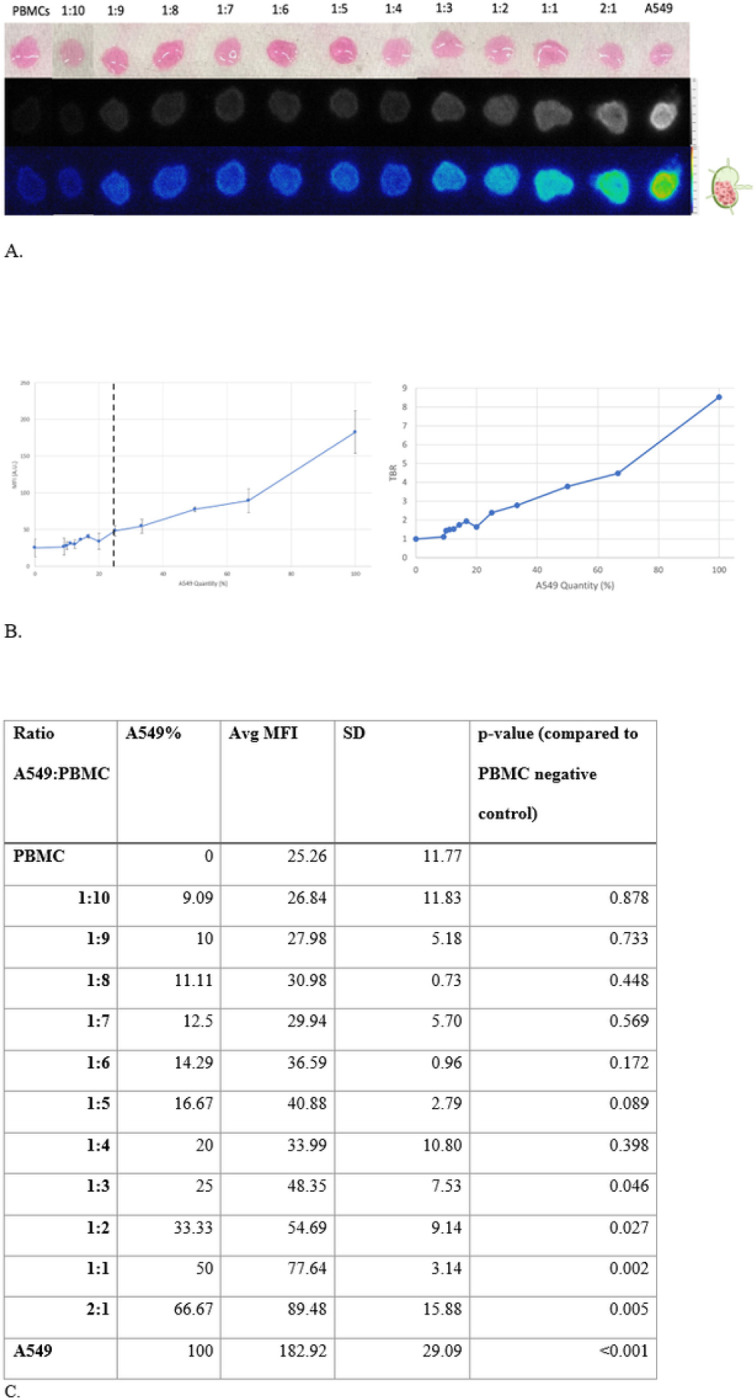
Lymph node model 1A for tumor replacing native parenchyma (A) 3D cell aggregates showing increasing ratios of A549:PBMCs while keeping total cell count constant. White light (top row), NIR monochromatic (middle row), and NIR heatmap (bottom row) images are shown. (B) (left)Minimum threshold for signal detection indicated by black dotted line. Ratios of 1:3 and greater (right-hand side of the dotted line) had significant fluorescence intensity (p<0.05). (right) Tumor to background ratios of the various MFIs(C) Independent sample t-test comparing the MFI of various ratios of A549:PBMC to the PBMC negative control.

Our controls included a 3D aggregate of PBMCs alone as a negative control (mean MFI = 25.26 ± 11.77 A.U.) and one of A549 alone as a positive control (mean MFI = 182.92 ± 29.08 A.U.). When cathepsin activity was found in 25% of the LN (mean MFI = 48.35 ± 7.53) to 66.67% (mean MFI = 89.48 ± 15.88 A.U.), the MFIs were greater than the PBMC negative control (p < 0.05). Hence, the minimum threshold for the detection of VGT-309 fluorescence in LNs by IMI is having 25% cathepsin activity. (Fig. 4)

###### b. Model 1B

**Figure 5:**
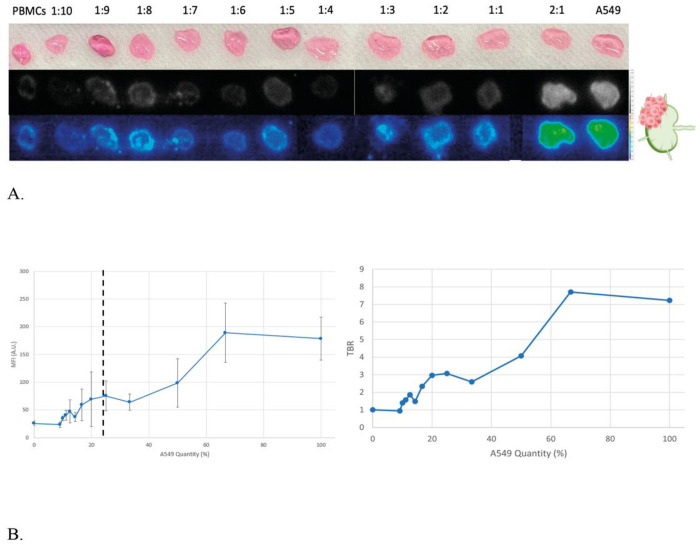
Lymph node model 1B for tumor growing adjacent to native lymph node parenchyma (A) 3D cell aggregates showing increasing ratios of A549:PBMCs while keeping PBMC count constant for each ratio. White light (top row), NIR monochromatic (middle row), and NIR heatmap (bottom row) images are shown. (B) (left) Minimum threshold for signal detection indicated by black dotted line. Ratios of 1:3 and greater (right-hand side of the dotted line) had significant fluorescence intensity (p<0.05). (right) Tumor to background ratios of the various MFIs

The minimum cathepsin activity threshold for signal detection compared to the PBMC negative control (mean MFI = 25.78 ± 4.45 A.U.) was 25% (mean MFI = 75.27 ± 26.96 A.U., p = 0.035). All ratios with greater percentages of cathepsin activity had significantly greater MFIs than PBMC alone (p < 0.05). (Fig. 5)

###### c. Model 1C

**Figure 6:**
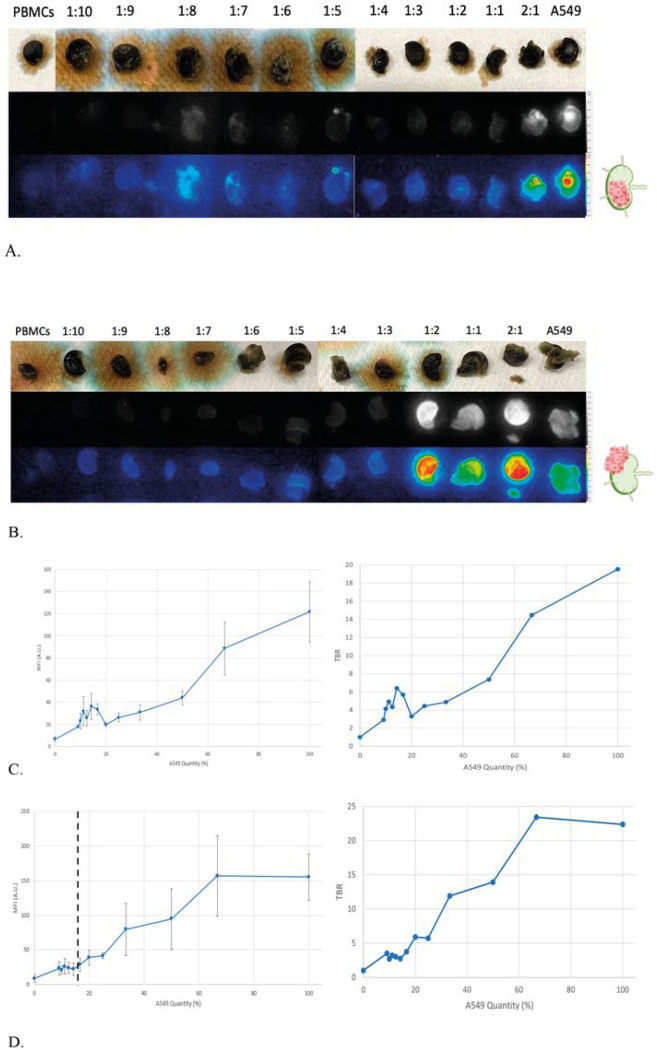
Ex-vivo model of anthracitic lymph nodes. (A) Model of an anthracitic LN in which tumor cells replace native parenchyma. All ratios have greater MFIs than the PBMC negative control (p<0.05) (B) Model of an anthracitic LN in which tumor cells grow adjacent to native parenchyma. Minimum threshold for significant signal detection is 16.67% of A549 quantity indicated by black dotted line (p<0.05). (C) (left) Any A549: PBMC ratio greater than the control had significant fluorescence intensity (p<0.05). (right) Tumor to background ratios of the various MFIs (D) Minimum threshold for signal detection indicated by black dotted line. A549: PBMC ratios of 1:5 and greater (right-hand side of the dotted line) had significant fluorescence intensity (p=0.03). (right) Tumor to background ratios of the various MFIs

To mimic anthracitic LNs, we added black food coloring to the previous two 3D models, 1A and 1B. Figure 6A resembles an anthracitic version of Model 1A in which total cell count (A549 + PBMC) was kept constant to represent tumor cell replacement of native parenchyma. The MFIs were significantly greater than the PBMC negative control (mean MFI = 6.721 ± 2.42 A.U.) even for a 9% cathepsin expression. (mean MFI = 17.936 ± 0.82 A.U., p = 0.002) Fig. 6B demonstrates anthracosis of Model 1B in which PBMC count is kept constant to represent the growth of tumor cells adjacent to native parenchyma. The minimum cathepsin expression for a significant increase in fluorescent signal detection was 16.67% (mean MFI = 28.78 ± 9.36 A.U., p = 0.03).

Tumor to background ratios of the various MFIs

##### Model 2 Testing with VGT-309: Inoculating Murine Spleens with VGT-309-labeled A549 cells

**Figure 7:**
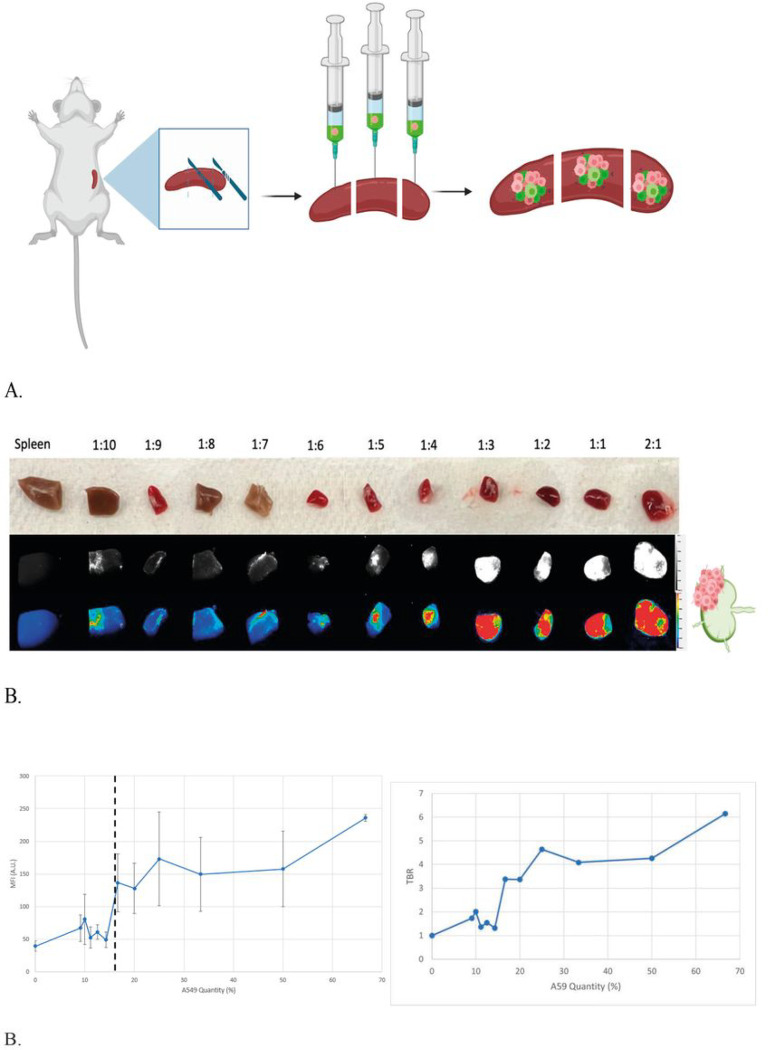
Ex-vivo model of positive LNs using murine spleens. (A) Murine model of sacrificed mice, spleen sectioned into 3 pieces, and injection of VGT-309 and A549 (B) Murine spleens with increasing ratios of A549:splenocytes to model tumor growth adjacent to native parenchyma. Spleens without A549 were used as negative controls. White light (top row), NIR monochromatic (middle row), and NIR heatmap (bottom row) images are (B) Minimum threshold for significant fluorescence signal detection (1:5) indicated by black dotted line (p=0.02).)left) (right) Tumor to background ratios of the various MFIs (right)

To test the ability of IMI to detection fluorescence in our spleen model, we injected the spleen segments with VGT-309-labeled A549 cells in increasing ratios (Fig. 7A). MFIs became significantly higher than the spleen negative control when cathepsin expression is increased in (mean MFI = 39.65 ± 8.13 A.U.) 16.67% of the cells.(mean MFI = 136.33 ± 44.55 A.U., p = 0.02) (Fig. 7B)

## Discussion and Conclusion

In this study, we describe the first two *exvivo* positive LN models. We then test these models for fluorescence detection using IMI. The first model is a 3D cell aggregate model with varying ratios of cancer cells and represents the two main ways cancers grow in LNs. One is contiguous to the lymph node parenchyma. The other is by replacing the LN parenchyma. As a variation of the first model, we added a black dye to create an anthracitic model. LNs in the lung for example can get stained by an anthracitic pigment secondary to pollution in the air or smoking.[18] The second model was based on the premise that the spleen is the largest lymphatic organ. A murine spleen was divided into three segments and various ratios of cancer cells were inoculated into it.

In our initial IMI experiment, we demonstrated that with a constant amount of dye, increasing the number of cancer cells, which correlates with increased cathepsin activity, increases the observed fluorescence. In other words, LN fluorescence and its brightness are correlated with cathepsin expression. We then translated that principle into our two LN models.

We observed that 25% of cells in a node need to have increased cathepsin activity to be detected by IMI with VGT-309 in the 3D cell aggregate model. Interestingly, the threshold to detect positive nodes through IMI in both anthracitic models was lower than in the models without food staining. We believe that this is partially due to a phenomenon called auto-fluorescence that may dampen the measured MFI and the signal observed. Our PBMC negative controls in the non-anthracitic, 3D cell aggregate model have a low but noticeable autofluorescence. Adding black food coloring dampens this auto-fluorescence and renders minute differences in fluorescence more detectable. As such, we are more likely to see a difference at a lower threshold in anthracitic nodes. Meanwhile, our second model had similar results to the lymph node with food stain and required 16.67% increased cathepsin activity to fluoresce significantly. These results are not surprising as the spleen can have hemosiderin deposits that can cause a change in color and behave similarly to anthracosis.[19]

The way cancer cells aggregate in LNs vary between different cancer subtypes. There are observations where all the cancer cells are clustered in one location. While that might be the case for some cancers, there is data that micrometastases are found as discontinuous disease in the LN.[20] One of the advantages of these two models is that they show both spectrums of LN topography. The 3D cell model assumes that micrometastases is disseminated throughout the node as the PBMCs and A549 cells are mixed and then solidified into the matrix. Meanwhile, the malignant cells in the murine spleen model are localized to the site of injection. As such, these models can be used to study different configurations of a positive node.

Intraoperatively, frozen section is used to identify suspicious nodes in certain malignancies. For instance, frozen section was used in sentinel LN biopsy (SLNB) but was rapidly abandoned due to the high false-negative rate. More modern imaging modalities include immunohistochemistry (IHC) in addition to traditional hematoxylin and eosin. IHC allows detection of microscopic disease at the single cell level.[21] IMI has helped in localizing tumor that is invisible to the naked eye and cannot be identified using tactile sensation.[9] It has also been applied to LNs but has not always been successful since patients at the time of surgery have typically undergone neoadjuvant therapy or have earlier disease stages, making it less likely to have enough tumor burden in the node to fluoresce avidly. Since there is no ideal way currently to quantify and identify positive nodes, relying on the society guidelines to address nodal dissection remains the most prudent approach. Regardless, when we do observe fluorescence in lymph node using VGT-309, it can indicate that at least 9% of the node is involved based on our study.

This study and model have certain limitations. First, LNs are dynamic and contain a flux of cells that always changes. This is a more static model and a snapshot of how lymph nodes would likely look *in vivo*. Also, for our purposes we used the murine spleen to mimic LN anatomy, but it is clearly different from a human LN. Thirdly, this study focuses on a dye that targets cathepsins, which can be overexpressed in tumor-associated macrophages.[22] In vivo, these macrophages can be found in LNs and may give a false positive. Lastly, this model uses the A549 cell line and VGT-309 as the fluorophore. Our study provides a model and framework for further that are needed to extrapolate these findings to different dyes and different cell lines.

This is the first exvivo positive LN model that was tested using IMI principles. Our findings suggest that a low tumor burden may result in false negative LNs that are missed by surgeons using IMI. In this study, we identified a minimum threshold for detection using cathepsin expression as an indicator. This threshold may differ depending on the NIR contrast agent, molecular target, and cell line used. Our models provide the foundation for more elaborate models that can be more dynamic in nature to reflect the actual lymph node microenvironment. Furthermore, this model can be used in testing different dyes ex-vivo and developing more sensitive imaging modalities for LN detection. We are currently conducting further studies that evaluate other cell lines and histologic subtypes with various dyes. Ideally, we hope these models will allow to localize micrometastatic disease at lower threshold, prevent recurrence, and improve survival.

## Data Availability

The data generated in this study are displayed in the manuscript. Data is available upon request from the corresponding author.
